# Facial vein injection of human cells in severe combined immunodeficiency (SCID) neonatal mice

**DOI:** 10.1016/j.mex.2018.10.006

**Published:** 2018-10-06

**Authors:** Nimshitha Pavathuparambil Abdul Manaph, Mohammed Al-Hawwas, Liang Liu, Donghui Liu, John Hayball, Xin-Fu Zhou

**Affiliations:** a School of Pharmacy and Medical Sciences, Sansom Institute, University of South Australia, Adelaide 5000, South Australia, Australia; b Central Northern Adelaide Renal and Transplantation Service, Royal Adelaide Hospital, Adelaide 5000, South Australia, Australia; c School of Medicine, Faculty of Health Sciences, University of Adelaide, Adelaide 5000, South Australia, Australia; d Jiangsu Key Laboratory of Drug Screening, China Pharmaceutical University, Nanjing, China

**Keywords:** Facial vein injection in P3/P4 pups, Neonates, Facial vein, Intravenous injection, Stem cells, Delivery

## Abstract

Intravenous injection is a standard procedure for delivering human stem cells and therapeutic agents. Currently, genetically modified severe combined immunodeficiency (SCID) mice are used for engraftment studies using human cells. SCID neonates have better integration and survivability of human cells compared to adult SCID mice, as their immune system will not be developed in the first few days after birth. However, intravenous injections in neonates are difficult. This protocol describes a reliable and reproducible method for injecting cells into the facial vein of P3/P4 (3 or 4 days post-birth) SCID neonates to study their engraftment. The injection was safe and well tolerated by the pups. Post-injection analysis revealed the distribution of tagged cells in different organs. Results suggest that this new method can serve as a pre-analysis for transplantation studies using human stem cells before *in vivo* animal model testing.

## Highlights

• Precise intravenous injection of human or mice stem cells in immunocompromised neonates.
• Fastest method of facial vein injection with confirmation of the procedure success.
• Direct method of stem cell delivery in neonatal brain, heart and liver.

## Graphical abstract

**Figure:**
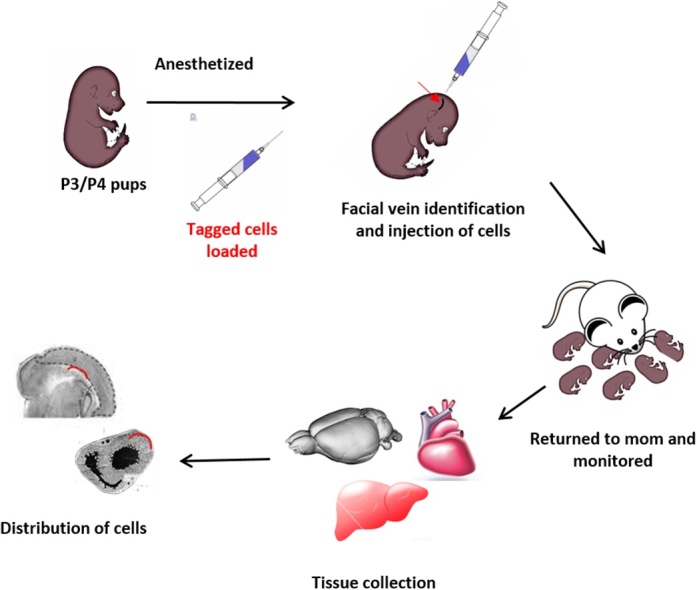

Specifications Table

**Table:** 

Subject area	• *Neuroscience* • *Medicine and Dentistry* • *Veterinary Science and Veterinary Medicine*
More specific subject area	*Intravenous injection*
Method name	*Facial vein injection in P3/P4 pups*
Name and reference of original method	*Gombash Lampe SE, Kaspar BK, Foust KD. Intravenous injections in neonatal mice. J Vis Exp.2014; 93*
Resource availability	*All reagents and instruments indicated are commercially available. Source of critical components are indicated in the manuscript clearly.*

## Method details

Stem cell research and pharmaceutical studies administer *in vivo* transplantations in animals to test the efficacy and survivability of stem cells or therapeutic agents. Subcutaneous and intravenous injections in rodents are universally accepted methods of testing the safety of stem cells [1]. Intravenous injections can facilitate the distribution of cells to different organs faster than subcutaneous injections and therefore, widely used [1]. Besides, intravenous injections are easy to perform in adult animals with minimal welfare impact.

Testing of human cells is generally carried out in Severe combined immunodeficiency (SCID) animals for cell therapy as their immune system are modified to provide better acceptance of cells without rejection [2]. SCID neonates have a better acceptance of human cells compared to adult mice, as their immune system will not be well developed for the first few days post-birth [3]. Intravenous injections have been demonstrated in P1/P2 SCID neonates successfully [4]. The temporal facial vein in SCID neonates are well visible during the first two days and allows reliable injection of cells during the time. However, the welfare impact of this procedure is high and in most cases, the mother rejects the pup leading to either the death of the animal or being eaten by the dam. From P3, the pups are bigger and the welfare impact will be less compared to P1 animals. Furthermore, intravenous injections on P3 animals are tricky, as the vein gets less visible than P1/P2; making the procedure more complicated. Here we describe an optimised method for injecting cells into the facial vein of P3/P4 neonates. The whole procedure takes less than 5 min and is safe, reliable and well tolerated by the animals. Furthermore, the method can be utilised to target the distribution of cells into different organs like brain, heart and liver (rather than the direct transplantation into those organs) to study the survivability and functionality, better than in adult mice. This method can also serve as a pilot study before testing the stem cells in *in vivo* animal models.

## Material and methods

### Animals

All procedures were pre-approved by the animal ethics committee of University of South Australia (ethics no: U06-17).

### Cell culture

We used human skin fibroblasts for the injections. The cells were firstly cultured in DMEM with 10% FBS and passaged for 5–6 times, then transfected with a Lentivirus harbouring a red fluorescent protein gene (RFP) at a concentration of 2.5 × 10^7^ particles at 10 mg/ml in 1 ml of culture media. Fresh media was added 24 h post transfection and the cells were visualised for RFP expression by live cell imager (ZOE Fluorescent Cell Imager, Biorad). Cells expressing RFP were sorted and harvested with a cell sorter (FACSAria II Cell Sorter, BD). The human skin fibroblast cell line expressing red fluorescent protein were allowed to expand until the procedure day. The cells were detached from the culture plates using the digestive reagent TrypLE (Thermofisher Scientific, VIC, Australia) and were resuspended in PBS. For each pup, 0.5–1 million cells in 50–100 μl volume were prepared.

## Experimental set up for injection

The procedure was performed on heat pad to ensure the animals receive proper warmth after separated from their mom. Aseptic procedure was followed (Fig. 2A).

**Fig. 1:**
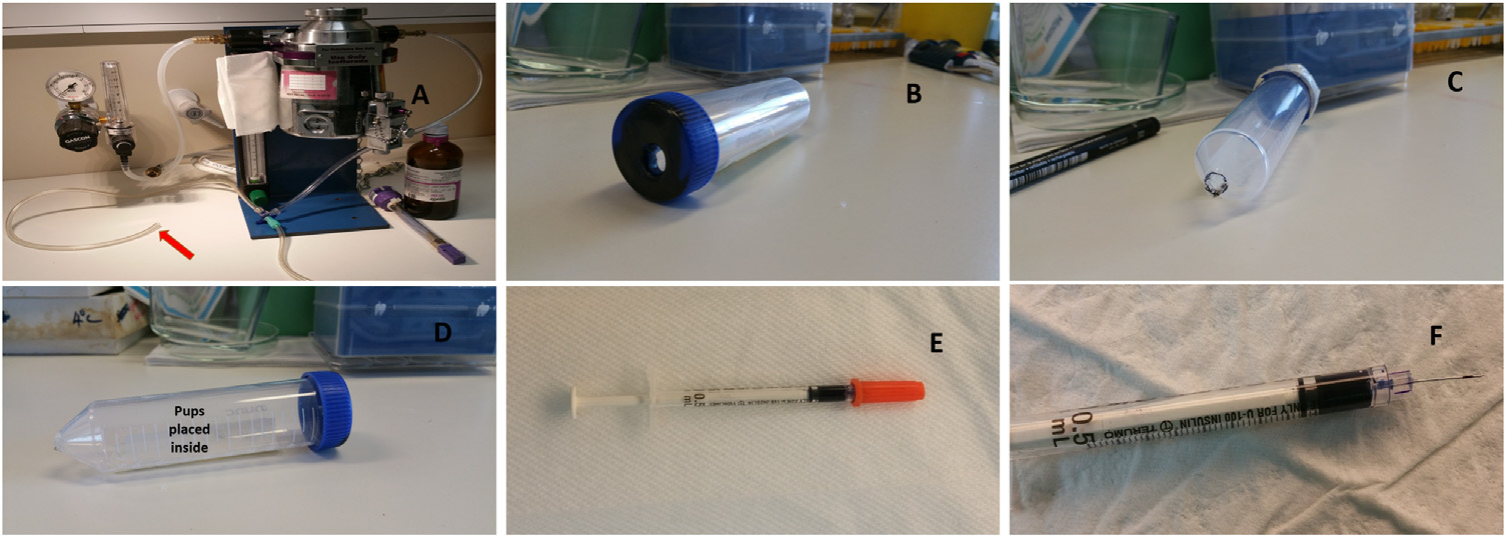
Anaesthesia and cell preparation. A. Anaesthetic machine. Arrow indicates the anaesthetic tube to be connected to the modified 50 ml tube with hole. B: Modified 50 ml tube with hole for connecting anaesthetic tube. C: Hole made at the rear end to avoid pressure from the flow of the anaesthetic gas. D: The pups were individually anesthetised in the container showed. E: The syringe loaded with cell (demonstrated using a blue dye). F: Marking made at the tip of the needle to monitor the injection to avoid deep insertion of the needle.

**Fig. 2:**
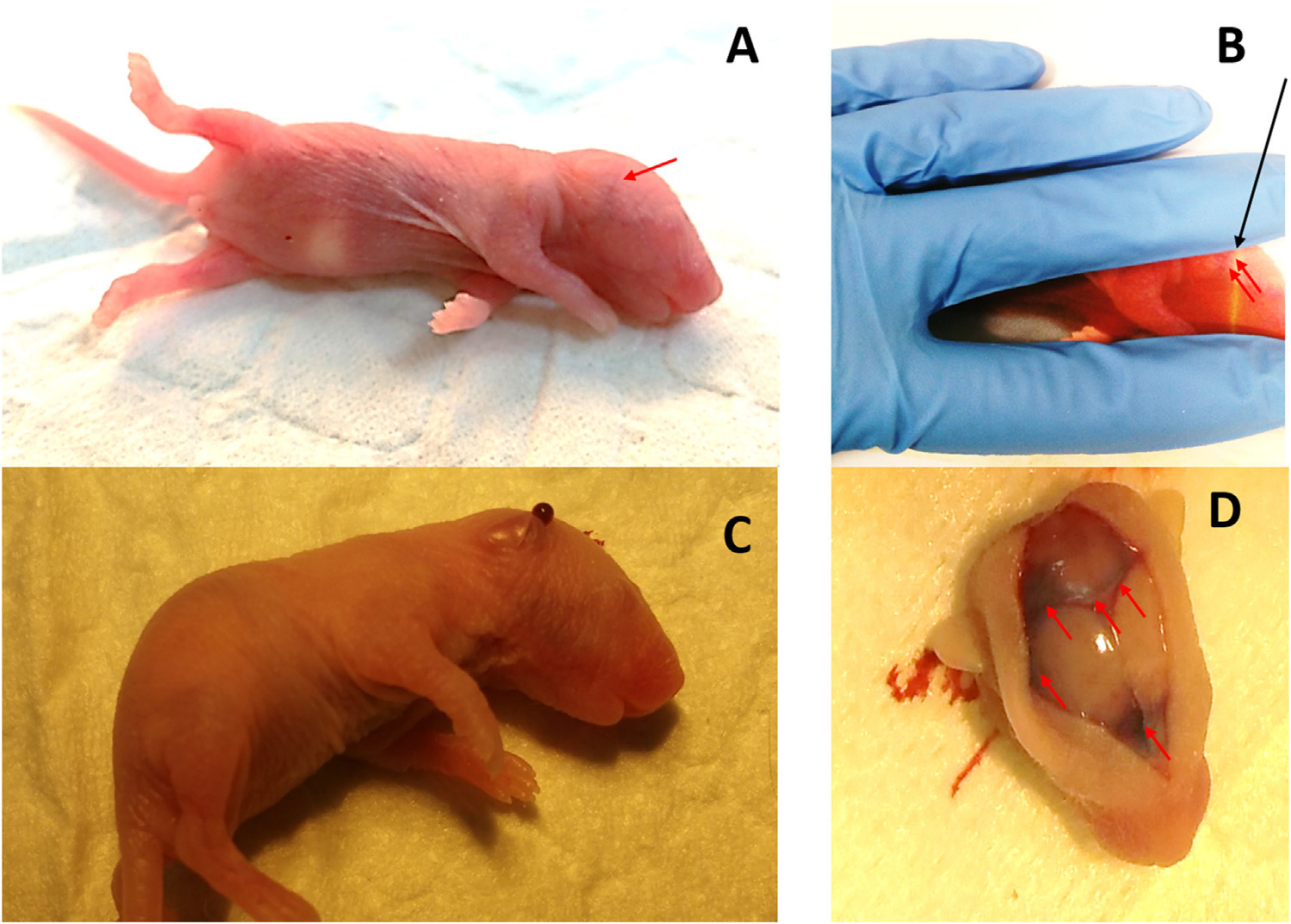
Key steps involved in the injection. A: Facial vein appearance in P3 pup. B: Holding the pups between the middle and index finger with the vein still visible (red arrow). Black arrow: The angle at which the needle has to be held for injection. C: Appearance of blood after inserting the syringe. D: Injection using blue dye demonstrated the presence in brain.

## Procedure

Neonates were anesthetised as follows:

1 The pups were individually placed in a modified 50 ml tube (Fig. 1D) with holes at both sides (Fig. 1B, 1C). The holes were made to allow the anaesthetic gas inside and for the passage of air in and out of the 50 ml tube to avoid the lid popping off with air pressure while the anaesthetic gas is passed through the tube.
2 The tube was closed and the anaesthetic tube containing isoflurane was connected through the big hole through the lid of the tube. The pups were placed inside the tube (Fig. 1D).
3 Pups were anesthetised with isoflurane (1.5–2.5%) in oxygen till they become immobilised. Generally, it takes 5–10 min for the neonates to get properly anesthetised.
4 Meanwhile, the 29 G insulin needle was preloaded with the cells (Fig. 1E). Mark the needle tip to avoid deeper insertion of the needle into the animal (Fig. 1F)
5 Once anesthetised, pups were placed on heat pads to perform the injection.

The injection was performed on the anesthetised neonates as follows:

1 The anesthetised animal was placed under the light source to view the partial vein near to the ear bud (Fig. 2A). For pups with partial vein appearance, the vein can be better localised if viewed under a magnifier or microscope.
2 For a right-handed person, the animal was held in a way that the vein can be more visible between the index and middle fingers (Fig. 2B).
3 After locating the vein, the area to be injected was wiped with ethanol (to sterilise and for better visibility of vein) and the syringe was faced at a 45° angle to allow precise insertion of the needle (Fig. 2B). Ensure the vein is still visible under the microscope after cleaning with ethanol.
4 The tip of the syringe was slowly inserted into the visible vein. If properly injected, it is possible to see blood through the skin (Fig. 2C). Allow 5–10 s to finish the injection and slowly remove the syringe to avoid the injectant coming out of the skin. Removal of the needle after the injection also causes oozing of blood from the vein.
5 The success of the injection can be easily identified with the bolus or non-bolus formation (bolus indicates the injection was not through the veins and non-bolus indicates the injection was successful).

## Post-injection care

Immediately after the procedure, the neonates were placed on a heating pad until they were recovered from the anaesthetic. Depending on the animal, it takes 20–30 min to recover completely from anaesthesia. Neonates were returned to their mother and covered with the cotton nestled so they will smell familiar to parents. The animals were monitored for acceptance by their mother, bruising around the injection site and mobility. Animals were kept alive for 3 days to one week and following that they were humanely killed for tissue analysis.

## Tissue collection and analysis

The humane killing was performed by pentobarbitone overdose and the tissues were collected by perfusion and fixation using paraformaldehyde. Brain, heart and liver were harvested for analysis. Organs were fixed in 4% paraformaldehyde for 18–21 h and immersed into 30% sucrose solution till tissue sunk. Free-floating staining was followed. The tissue was placed on cryomold overlayed with optimal cutting temperature (OCT) compound. Once frozen, it was properly oriented and sections were cut at 15–30 μm using cryostat (Leica CM3050 S Research Cryostat, Mannheim, Germany). Cut sections were transferred to antifreeze solution. For analysis, the cut sections were washed two times with 1xPBS and mounted onto slides using coverslips and analysed using fluorescent microscope (instrument details).

## Results and discussion

The key step in the above procedure is to identify the veins correctly during the injections. Veins were only partially visible in P3/P4 pups, therefore, the procedure was performed under a magnifier. Cleaning the injection site with ethanol further increases the visibility of the vein. With practise, we were able to perform and optimise the procedure to ensure successful facial vein injection on 16 (n = 10 using blue dye to master the method and n = 6 with stem cells), P3/P4 pups. It is also equally important not to insert the needle too deep through the vein, which can result in partial or unsuccessful injection. Successful injections can be confirmed by the non-bolus formation post-injection. A partial or unsuccessful injection can be identified by the bolus formation around the injected area. However, on monitoring, the cells were absorbed into the body within 20–30 min and resulted in normal appearance of the animals. If the vein is properly located, with steady hands, up to 100 μl volume of resuspended cells can be injected. There was no bruise or inflammation around the injection site for this procedure. In addition, we performed the anaesthesia using isoflurane rather than the ice slurry (hypothermia method) reported in the literature [5]. The injection procedure was performed successfully and had reduced welfare impact on the animals, as it took only 15–20 min for the whole procedure (5–10 min for anaesthesia and 5 min for the injection procedure under anaesthesia). Following the procedure, the animals recovered within 20–30 min and were returned to their mom.

**Fig. 3:**
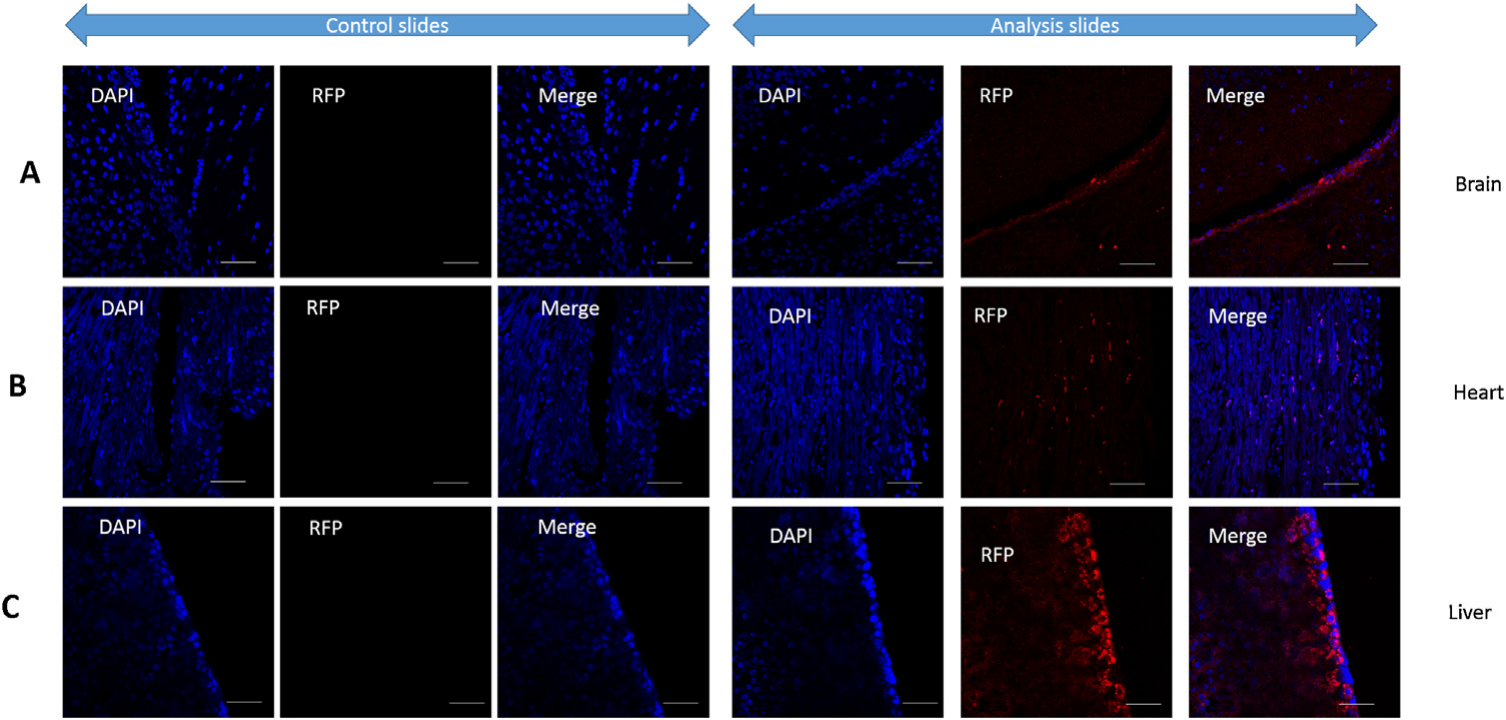
Tissue analysis for tagged cell identification. A. Tagged cells identified in control (saline injected) and stem cell injected brain. B: Tagged cells identified in control (saline injected) and stem cell injected heart sections. C: Tagged cells identified in control (saline injected) and stem cell injected liver sections.’ DAPI’ indicates individual nuclear staining in the samples. ‘RFP’ represents the red fluorescent protein tagged cells in the section and ‘Merge’ indicates the overlay picture of DAPI and RFP picture. All pictures taken at 40X magnification. Scale bar: 100 μm, Representative figure from n = 3 injected mice organs.

Tissue analysis demonstrated the presence of tagged cells in brain, heart and liver (Fig. 3). However, we did not perform functional analysis on the tagged cells as we were interested only in the injection procedure and the distribution of cells in the aforementioned organs. Nevertheless, we do not expect fibroblasts to be functional in brain, heart or liver. In addition, we also expect the cells may have migrated to other organs such as lungs and spinal cord. Detailed analyses need to be carried out to understand the migration of cells into other organs.

## Conclusion

In this paper, we present an optimised method for facial vein injection in neonates using human stem cells. Single person can perform the injection within 5 min without unexpected adverse events from handling or the procedure. The injection was safe and well tolerated by the pups. In addition, the procedure caused the procedure caused distress only for a short time during the whole handling. Nevertheless, the procedure does not involve any restraining as in the intravenous injection of an adult mouse. However, detailed tissue analysis needs to be carried out to understand the functionality of the distributed cells and also the bio-distribution of cells in other organs like lung, spinal cord, spleen and pancreas for different stem cells. Although the method describes the procedure in SCID neonates, we expect this can be carried out in other mice breeds with less darker skin. Currently, drug designing incorporates extensive screening and testing of critical compounds. With functional analysis, this method can be also very well served as a quick alternative to screen therapeutic molecules on a smaller level.

## Funding

University of South Australia (UniSA), D&R Pharmaceutics China.

## Conflicts of interest

The authors indicate no conflict of interest.
